# Editorial: Native yeasts: isolation, characterization, and food industry applications

**DOI:** 10.3389/ffunb.2026.1822445

**Published:** 2026-04-13

**Authors:** Lambros Farmakis, Athanasia Koliadima

**Affiliations:** 1 Department of Food Science and Technology, University of the Peloponnese, Kalamata, Greece; 2 Department of Chemistry, University of Patras, Patras, Greece

**Keywords:** fermentation, metabolomics, native yeasts, starter cultures, yeasts biodiversity, yeasts functional properties

Yeasts represent one of the most important categories of fungi with plenty applications on human food systems. *Saccharomyces cerevisiae*, especially, has been used as a model organism and industrial powerhouse, and also for investigating – among others – native and unconventional yeast species as sources of biodiversity, metabolic innovation, and terroir-related functions. The Research Topic *“Native Yeasts: Isolation, Characterization, and Food Industry Applications”* gathers contributions that together enhance our comprehension of native yeast diversity, its molecular and phenotypic analysis, and its practical applications in various food matrices.

The main goal of this Topic is to illuminate the connection between microbial ecology and practical food biotechnology. Microbial communities of native yeasts with different origin—vineyards, orchards, cereal sources, dairy settings, natural fermentations, and various ecological niches— are influenced by several parameters such as geography, climate, plant genetics, and human activities. The research of these species requires not only traditional microbiology, but also novel approaches like high-throughput sequencing, omics-centered phenotyping, and systems-level fermentation analysis.

Several research papers deal with extraction and classification of native yeast strains from particular ecosystems. These studies link yeast biodiversity to food-related environments by connecting culture-dependent methods with molecular techniques. They aim to highlight the ecological uniqueness of yeast communities and the selective influences imposed by their local environments. These results support the idea of “microbial terroir,” where the role of native microbial communities on the sensory and functional characteristics of fermented products is of great importance.

This Topic aims not merely at classification, but primarily at the characterization of the functional properties of specific yeast strains, such as fermentative capacity, tolerance to extreme conditions (ethanol, osmotic pressure, temperature), enzymatic activities (β-glucosidase, protease, lipase), killer traits, biofilm formation, and profiles of metabolite production. The integration of phenotypic screening with metabolic analyses, alongside the parallel evaluation of volatile compounds, demonstrates that this Section successfully links the unique strain-specific metabolic “signatures” to the sensory attributes of the final products. This comprehensive approach is crucial for transitioning from simple strain isolation to their evidence-based selection for industrial exploitation.

Published articles clearly demonstrate the influence of non-*Saccharomyces* yeasts on fermentation dynamics and on the quality of final products. Genera such as *Hanseniaspora*, *Metschnikowia*, *Torulaspora*, and *Pichia*, which were previously regarded as insignificant spoilage microorganisms or transient early colonizers, are now recognized for their ability to enhance aromatic complexity, modulate acidity, reduce ethanol levels, or provide bioprotective effects in co-inoculation or sequential fermentation strategies. The findings presented in this Topic provide empirical support for the strategic integration of these native strains into controlled fermentations, challenging the dominance of monocultures and conventional starter cultures.

Another achievement of this Topic lies in highlighting the growing interest of industrial research in the exploitation of native yeasts, particularly given that some strains exhibit antagonistic activity against spoilage microorganisms and mycotoxin-producing fungi. These findings align with the increasing demand for “clean-label” preservation strategies and for reducing the use of chemical additives in food production. The application of native yeasts in food processing is fully consistent with contemporary industry requirements and consumer priorities.

The application of omics technologies is a common methodological approach across almost all contributions in order to analyze strain-level variability. For elucidating the molecular determinants underlying favorable phenotypes, multi-omics approaches, including genomics, metabolomics, and proteomics, were applied. Comparative genomics was used for the investigation of the relation between genes and stress tolerance and aroma compound production, while regulatory responses under industrial conditions were evaluated by transcriptomic studies during fermentation. The exploitation of such mechanistic knowledge enables the development of predictive models and the design of improved, tailor-made starter cultures suitable for specific substrates and processing conditions.

Notably, the published studies also emphasize the dual role of native yeasts both as technological tools and as integral members of complex microbial communities. Interactions between yeasts, as well as between yeasts and bacteria in mixed fermentations, are investigated through mechanisms such as metabolic cross-feeding, competition, and synergistic interactions, which are considered critical for shaping fermentation kinetics, product stability, and sensory characteristics.

The significant industrial application potential of native yeasts constitutes another key parameter highlighted in this Section. Studies conducted in wine, beer, cider, bread, dairy products, and other fermented foods reveal common behaviors and challenges across different substrates. The use of native yeasts appears to represent a valuable strategy for enhancing aromatic diversity, improving nutritional value, reducing undesirable metabolites, and strengthening process reliability.

The main conclusions arising from the present Topic can be summarized as follows: (a) the need for new yeast strains with distinct metabolic capabilities is ongoing, and their exploration takes place in both well-studied and emerging ecosystems; (b) the transition from biodiversity to practical application requires in-depth phenotypic and genotypic characterization of yeasts; and (c) the strategic exploitation of native yeasts—either as single-strain starter cultures or as components of designed microbial consortia—offers substantial opportunities for the production of innovative products, the enhancement of sustainability, and the improvement of safety in the food sector.

More broadly, the conclusions of the published studies reinforce the prevailing view that fermentation can function as a regulatory factor in the production of fermented foods. Native yeasts may be regarded as the critical link between the environment, raw materials, and technological intervention. The study and utilization of their role in food biotechnology emerge through a multidimensional and multifactorial approach that integrates microbial ecology, molecular biology, food chemistry, and process engineering.

In closing, we would like to thank all the authors and reviewers who contributed to this Topic, without whom the successful completion of this effort would not have been possible. We hope that this Topic will serve as a starting point for further exploration of yeast biodiversity, will strengthen interdisciplinary collaborations, and will accelerate the transfer of fundamental knowledge into the development of innovative and sustainable industrial practices in food production.

